# Vulnerability to predation may affect species distribution: plovers with broader arctic breeding range nest in safer habitat

**DOI:** 10.1038/s41598-020-61956-6

**Published:** 2020-03-19

**Authors:** Don-Jean Léandri-Breton, Joël Bêty

**Affiliations:** Département de Biologie et Centre d’Études Nordiques, Université du Québec à Rimouski, 300 allée des Ursulines, Rimouski, QC G5L3A1 Canada

**Keywords:** Behavioural ecology, Biodiversity, Biogeography

## Abstract

Lower vulnerability to predation should increase the capacity of prey populations to maintain positive population growth rate in regions characterized by high predation pressure. Some arctic-nesting shorebirds nest almost exclusively in areas where predation pressure is regularly released. The few species that can breed within the entire distribution range of the Arctic Fox, the main nest predator in the arctic tundra, are supposedly less sensitive to predation. However, empirical data supporting this hypothesis are scarce and mechanisms driving interspecific variation in vulnerability to nest predation are poorly documented. We monitored nest success of two arctic-nesting shorebirds with contrasting breeding distribution and nesting habitat. We found that (i) when co-existing at the same breeding site, the widely distributed Ringed Plovers nesting along stony shores showed a higher nest survival rate than the Golden Plovers nesting in mesic tundra, and (ii) such differences in nest survival were at least partly driven by the nesting habitat type *per se*, with lower predation risk in stony shores than in adjacent mesic tundra. We suggest that the use of safer nesting habitat by some shorebird species can contribute to maintaining viable breeding populations over a broader distribution range.

## Introduction

The importance of biotic interactions in shaping species distribution and range limits is still highly debated^1^, and their effects at broad spatial scales are often hard to distinguish from abiotic factors like climate^2,3^. Moreover, the effects of biotic factors such as competition, facilitation and predation are difficult to assess because they typically involve complex interspecific interactions^4^. As defined by the niche concept, a species’ range should reflect the geographical space where the set of environmental factors (abiotic and biotic) corresponds to the species’ niche requirements, permitting persistence over time^5^. Predation is an important aspect of a species’ realized niche, affecting the capacity to maintain a positive population growth rate through additional mortality^6,7^. In ground-nesting birds, predation is a major cause of reproductive failure, and can have important consequences on bird population dynamics and life-history traits^8–10^.

**Figure 1:**
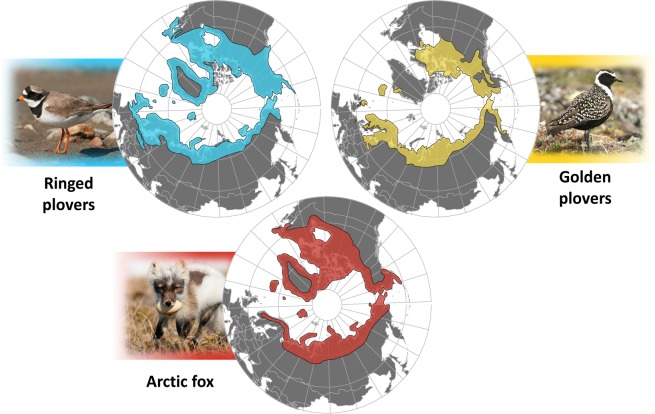
Breeding distributions of Ringed Plovers (Common Ringed and Semipalmated Plovers) and Golden Plovers (American, European and Pacific Golden Plovers) in comparison with the distribution range of the Arctic Fox. Note the wide distribution of Ringed Plovers, virtually overlapping the entire Arctic Fox distribution, and the absence of Golden Plovers from regions where Ringed Plovers are present. Data adapted from Cramp and Simmons^45^, IUCN^12^, Mehlum^59^ and Chester^57^. Arctic- centred maps based on orthodromic projection. Photo credit: D.-J. Léandri-Breton.

The Arctic Fox (*Vulpes lagopus*) is the main nest predator in the arctic tundra and has a wide circumpolar distribution^11^ (Fig. 1). Some shorebird species are able to breed across most of the Arctic Fox’s distribution while others are absent from large regions^12^. Gilg and Yoccoz^13^ suggested that shorebird species that can breed within the entire distribution range of the Arctic Fox are less sensitive to predation. For shorebird species that are most sensitive to predation, viable populations would occur only in areas where the predation pressure imposed by the Arctic Fox is lower or regularly released, thus restricting their nesting distribution^13,14^. Evidence supporting this hypothesis comes from species distribution patterns (mismatch and co-occurrence of species) and not from demographic parameters, which are essential to fully identify the main drivers of species distribution^1^. Moreover, the proximate mechanisms generating interspecific variation in vulnerability to predation remain poorly studied in tundra-nesting shorebirds, but incubation behaviour and nesting habitat are likely key factors generating differences among species^15–17^. More conspicuous incubation behaviour (e.g., frequent incubation recesses) is indeed generally associated with higher risk^15,16^ and habitat structure can affect risk of predation in the arctic tundra^18^. Investigating the relative vulnerability to predation of tundra-nesting shorebirds with contrasting geographical ranges, differing in extent and overlap with their main predators, should help evaluate the potential role of predation in shaping their distribution.

**Figure 2:**
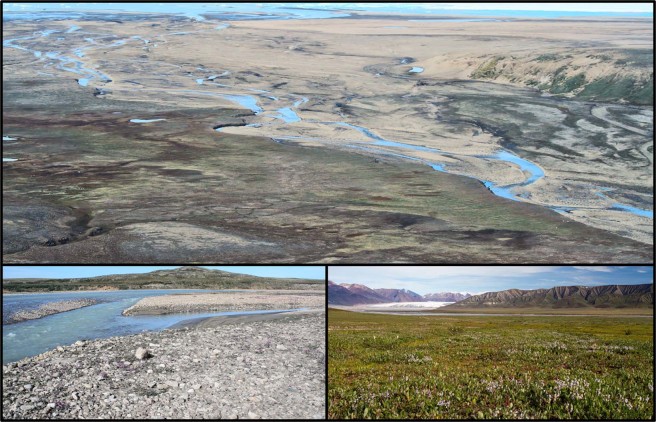
Photos taken at the study site located in the Canadian Arctic and showing two contrasting but adjacent habitats used by nesting plovers. Stony shores (bottom left) found along rivers are the main nesting habitat of the Common Ringed Plover (*Charadrius hiaticula*) while the American Golden Plover (*Pluvialis dominica*) breeds mainly in mesic tundra (bottom right). Photo credit: D.-J. Léandri-Breton (top and bottom left) and Nicolas Bradette (bottom right).

We tested the hypothesis that arctic-nesting shorebirds that can persist over larger breeding distribution range, such as Ringed Plovers (i.e., the sister species Common Ringed Plover *Charadrius hiaticula* and Semipalmated Plover *C. semipalmatus*; Fig. 1), are less vulnerable to nest predators than plovers with more restricted breeding distributions such as the Golden Plovers (i.e., the sister species American Golden Plover *Pluvialis dominica*, European Golden Plover *P. apricaria* and Pacific Golden Plover *P. fulva*; Fig. 1). We also tested the hypothesis that differences in vulnerability to nest predators is partly driven by the nesting habitat used by plovers, with Ringed Plovers nesting in safer habitat than Golden Plovers. The distribution of Ringed Plovers extends over a wider latitudinal gradient (4500 km, 43°–83° N) than that of Golden Plovers (2800 km, 52°–77° N) and covers large areas where the latter are absent (e.g., South and West Greenland, Svalbard, temperate regions of Eastern North America and Western Europe^12^; Fig. 1). Tundra-nesting Ringed Plovers and Golden Plovers are both long-distance migrants with circumpolar distribution and their breeding ranges are not restricted by major ecological barriers. They are both biparentally incubating plovers^16^ and they exhibit similar distraction displays and anti-predator behaviours at their nest^19^ but use distinct nesting habitat. We predicted that when co-existing at the same breeding site, the Ringed Plovers, nesting mainly in stony shores, would be less vulnerable to nest predators than the Golden Plovers, nesting mainly in mesic tundra (Fig. 2). We quantified nest survival rate of Common Ringed and American Golden Plovers in the Canadian High Arctic and conducted field experiments with artificial nests to quantify predation risk in the main nesting habitat used by each species. A lower predation risk in the main nesting habitat used by the Common Ringed Plover would indicate that such species are more likely to maintain a positive population growth rate in regions characterized by high predation pressure.

## Results

Over three summers (2014–2016), we monitored Common Ringed Plover and American Golden Plover nests (n = 55 and n = 99, respectively) to estimate nest survival rates in the High Canadian Arctic. Daily nest survival was significantly higher for the Common Ringed Plover than for the American Golden Plover (logistic exposure model, χ^2^(1) = 28.6, estimate coefficient 2.1, SE 0.5, *p* < 0.001), and the difference was similar for all years (GLM testing for species-year interaction, χ^2^(2) = 0.1, *p* = 0.9). The annual mean daily survival rate over the 3 years of the study was 0.994 (±0.001 SE, min = 0.991 and max = 0.996) for the Common Ringed Plover and 0.952 (±0.005 SE, min = 0.946 and max = 0.961) for the American Golden Plover. Over the entire incubation period, it represented an average success rate of 86% (±0.03 SE) for the Common Ringed Plover and 28% (±0.04 SE) for the American Golden Plover. The mean clutch size was 3.8 eggs (±0.06 SE, n = 65) for the Common Ringed Plover and 3.9 eggs (±0.03 SE, n = 115) for the American Golden Plover. For successful nests, the mean number of hatched eggs was 3.9 (±0.1 SE) for both Common Ringed and American Golden Plovers (n = 29 and 35 nests, respectively).

**Figure 3:**
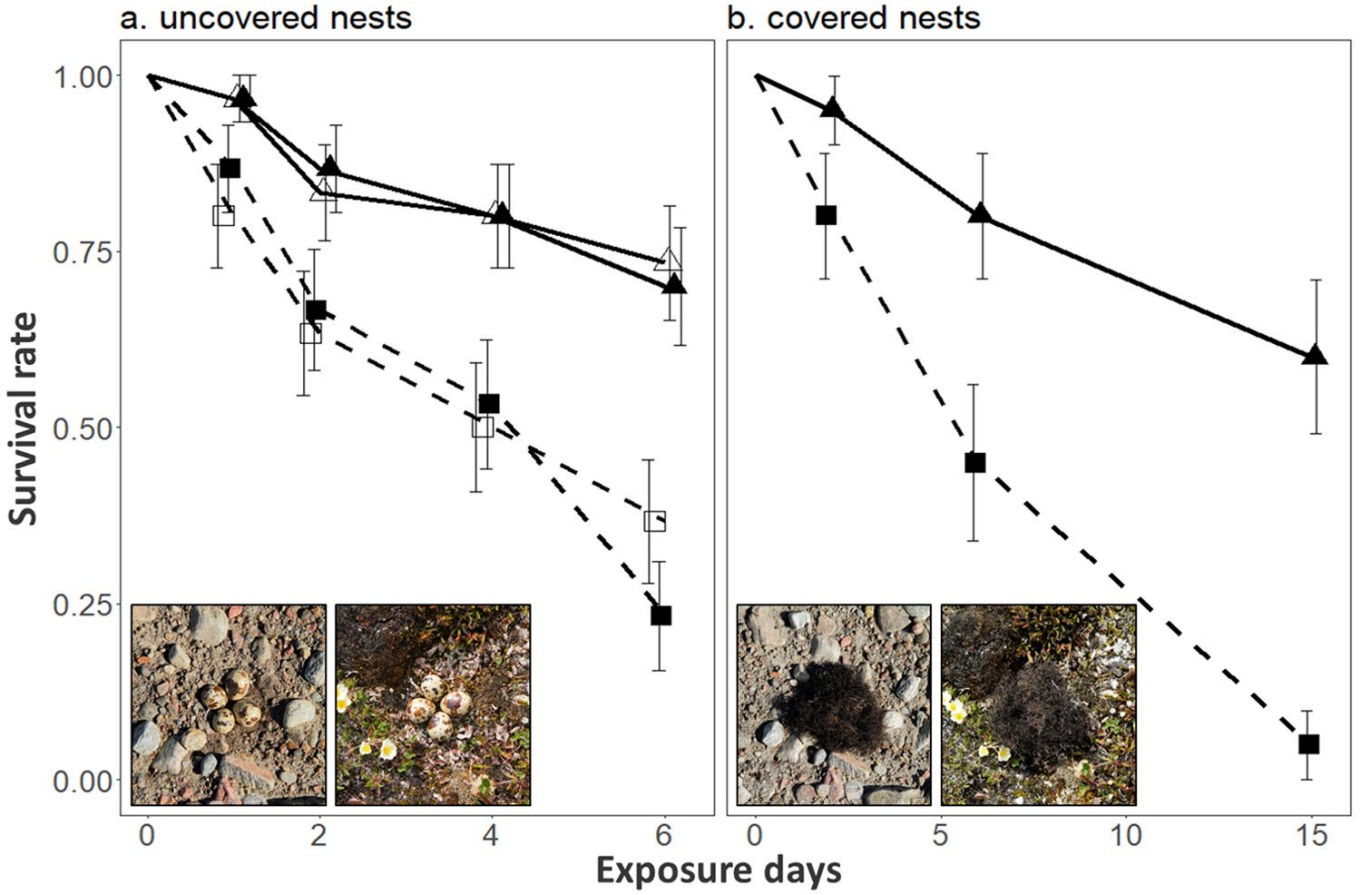
Kaplan-Meier survival probabilities for uncovered (**a**) and covered (**b**) artificial nests deployed in stony shores (triangles; solid lines) or mesic tundra habitat (squares; dotted lines) in the Canadian High Arctic. Each data point represents the survival estimate at time *t* (with mean standard error). The experiment with uncovered nests was repeated two years (2015: empty symbols; 2016: filled symbols).

Common Ringed Plover and American Golden Plover nests were found in very distinctive breeding habitat (stony shores and mesic tundra, respectively), which are often adjacent to one another in the study area (Fig. 2). Artificial nests were used to assess predation risk in these two habitat types, as this allows us to control for potential sources of heterogeneity associated with real nest survival rate^20^, such as parental investment in anti-predator strategies^21^, degree of parental care^15^ or nest age^22^. We conducted two experiments, one using uncovered artificial nests, which are depredated by foxes and avian predators^23^, and a second experiment using artificial nests covered with a patch of lichen, which are depredated only by foxes in our study area (*see methods*). We found that habitat type had a significant effect on the predation rate of uncovered artificial nests (χ^2^(1) = 20.8, *p* < 0.001), with a survival rate 71% higher in stony shores than in mesic tundra habitat (Cox proportional hazards regression, *β* = −1.2, SE 0.3, χ^2^(1) = 18.5, *p* < 0.001, hazard ratio = 0.29; Fig. 3a). Predation rate was similar in both years (σ^2^ < 0.001, *p* = 0.95). The same habitat-driven pattern in predation risk was detected using covered artificial nests, with the survival rate being 78% higher in stony shores than in mesic tundra habitat (Cox proportional hazards regression *β* = −1.5, SE 0.4, χ^2^(1) = 13.9, *p* < 0.001, hazard ratio = 0.22; Fig. 3b).

## Discussion

We hypothesized that arctic-nesting plovers persisting over most of the Arctic Fox’s distribution range are less vulnerable to predation and that habitat-driven differences in nest predation risk could partly explain differences in vulnerability. As predicted, plovers nesting in stony shores and having a widespread breeding range showed much higher nest survival rates than plovers with a more restricted breeding distribution and nesting in mesic tundra. Using artificial nests, we confirmed that such differences in nest survival were at least partly driven by the nesting habitat type *per se*. Moreover, as we found the same habitat-driven predation pattern using uncovered or covered artificial nests, we conclude that reduced predation risk in stony shores was not caused by eggs being harder to find for predators in this specific habitat (i.e., egg crypsis^24^) but more likely due to a reduced Arctic Fox foraging effort in stony shores. The lower predation risk in the main nesting habitat used by the Common Ringed Plover likely increases the capacity of such species to maintain a positive population growth rate in areas characterized by relatively high predation pressure, such as waterfowl nesting areas^25^, and presumably lemming-free areas in the Arctic^13^ (see below).

The safer stony shores are likely less attractive for foxes, and our results showed that such habitat can provide a partial refuge for tundra-nesting birds at the local scale. In heterogeneous environments, optimal foraging models predict that predators should spend more time and more foraging efforts in patches with higher prey densities^26^. Harsh stony, sandy habitats are typically characterized by scarce vegetation with few potential prey for tundra predators like foxes. In our study area, the Common Ringed Plover is the only stony shore specialist^27^. Conversely, the American Golden Plover nests in mesic tundra where several tundra prey can be found, including waterfowl and small rodents^25,28^. The maximum plover nest density found in stony shore habitat is 0.10 (±0.03 SE) nests per ha, while goose nest density can reach up to 6.7 (±0.2 SE) nests/ha^29^ and lemming density can be as high as 5.7 (±0.4 SE) individuals/ha^30^ in the adjacent mesic tundra habitat. There are potential trade-offs to nesting along river shores habitat and birds using that strategy may face some costs, such as a risk of nest flooding following heavy rains and fast snow melt^31^. For instance, terns and shorebirds nesting on small low-level islands can benefit from the absence of mammalian predators but can face occasional flooding negatively affecting nest success^32^. Nesting in such habitat may thus require specific adaptations.

The Arctic Fox is the main predator of arctic and subarctic bird nests and, therefore, its fine-scale foraging habits across discrete habitats likely have substantial impacts on the distribution and abundance of prey species^14,33^. Within an ecological time scale, key predator species can affect the distribution of other species by continually driving populations of some species extinct while co-existing with others^34^. Arctic birds adapted to nesting in less risky habitat such as stony shores can likely persist better in regions characterized by a higher predation pressure. Nest predation typically leads to total clutch lost and partial clutch predation is rare in our study area (recorded in only 8 out of 169 depredated plover nests). In such case, high nest predation rate can have marked negative effect on bird fecundity and hence affect local population persistence^8–10^. However, breeding parameters such as nestling growth and survival can also affect population growth rate. Birds may face trade-offs between the quality of incubating and chick-rearing habitats. For instance, a safe nesting habitat could increase nest survival but reduce chick survival if food resources are too scarce.

Some arctic-nesting shorebird species nest almost exclusively within the distribution range of lemmings^13^. In lemming-free regions such as Southwest Greenland and Svalbard, densely vegetated mesic tundra is widely found in non-glacial lowlands^35,36^ and can support large populations of herbivores (i.e., goose nesting colonies and reindeers^36^). This suggests that the absence of some shorebird species in these extensive regions, such as the Golden Plovers, cannot be explained by the availability of mesic tundra *per se*. In areas devoid of lemmings, Arctic Foxes have a highly diversified diet and are generally more dependent on abundant localized prey such as bird colonies or carcasses of large mammals^37,38^. When lemmings are present, predation pressure imposed by the Arctic Fox on ground-nesting birds is regularly released and their breeding productivity enhanced in years of relatively high lemming density^39–43^. Arctic Fox’s diet is then largely specialized on lemmings^37^. Based on the assumption that nest predation pressure is higher on average and rarely released in areas devoid of lemmings, Gilg and Yoccoz^13^ suggested that shorebird species that are most sensitive to predation can occur only in areas with lemmings. However, there is yet no empirical evidence indicating that predation risk is higher in regions devoid of lemmings, and this would deserve further investigations.

Like the Common Ringed Plover, other shorebird species could benefit from a reduced predation pressure by selecting nesting habitat less attractive for foxes and hence have a wider distribution range. For instance, the Purple Sandpiper (*Calidris maritima*), which uses coarse gravel-sand beaches along rivers and barren rocky ridges and plateaus^44,45^, also breeds in large lemming-free areas (i.e. West and South Greenland, Svalbard and Franz Josef Land). Moreover, nest predation risk increases with decreasing latitudes in Eastern Canada^46^. Therefore, we would predict that habitat characterized by a low prey density and acting as partial refuge, like the stony shores, could be crucial for shorebirds nesting in southern regions. Interestingly, the most widespread North American shorebird, the Killdeer (*Charadrius vociferus*), nest on sandbars, barren grounds or gravelled fields^47^. Also, the other Eastern North American plovers, namely the Piping^48^ (*Charadrius melodus*), Snowy^49^ (*Charadrius nivosus*), Wilson’s^50^ (*Charadrius wilsonia*) and Collared Plovers^51^ (*Charadrius collaris*) are nesting on sandbars, gravel shores and pebble or sandy beaches, which likely represent habitats less attractive to mammalian predators.

Like stony shores, wet meadows may also be less attractive foraging habitat for foxes because prey like lemmings are typically absent or found in lower density in these habitats^52^, and because wetland areas can hamper the movement of mammalian predators while searching for prey^18^. Wet sedge meadows are associated with the lowest predation risk of five arctic shorebirds nesting habitats as measured with artificial nests, although this pattern was not confirmed with real nests^17^. The Red-necked Phalarope (*Phalaropus lobatus*) is an example of an arctic shorebird nesting in very wet graminoid meadows^53^ and it has a large circumpolar distribution including lemming-free areas^12,45^ (e.g. West and South Greenland, Svalbard). Interestingly, wet meadows, peats and other wetlands are the main nesting habitat used by shorebirds in temperate regions^45^. Overall, we suggest that habitat-driven predation risk may have a profound effect on shorebird distribution in temperate, subarctic and arctic regions.

Our study was conducted during years of moderate to high lemming density (see *Methods*), which typically results in a lower predation pressure on shorebird nests on Bylot Island^39^. Effect of habitat on nest predation rate is typically less pronounced during years of high lemming abundance^18^. We nonetheless detected a marked difference in nest success between the two selected plover species. On the other hand, the large snow goose colony present at our study site contributes to maintaining higher predation pressure on bird nests^25^. Although we used multi-year data, it would be highly relevant to investigate predation risk at several study sites to confirm that the habitat-driven differences found in our study are widespread, notably in regions where predation risk is expected to be higher on average, such as lemming-free regions^13,14^. It would also be relevant to test if other nesting habitats such as wet meadows could be used as partial refuge against predation for other widely distributed bird species.

Spatial heterogeneity in predation risk results in refugia that can have important positive effects on prey persistence over time^54^. Because predators tend to aggregate in high prey density patches, habitats associated with little prey availability could provide partial refuge, as they are less attractive to predators^55^. Our results showed that habitats characterized by low prey availability for Arctic Foxes, such as stony shores, can serve as partial refuge against predation at the local scale. Hence, species adapted to nest in such habitat would likely have an increased capacity to persist in arctic regions characterized by higher predation pressure. As suggested by Larson^14^ and Gilg and Yoccoz^13^, the absence of some arctic shorebird species in specific Arctic regions could be partly explained by their higher vulnerability to predation. Our study provides evidence indicating that some species with greater breeding ranges are indeed less vulnerable to predation because they use safer nesting habitat, which is in accordance with the hypothesis that vulnerability to predation could affect species distribution ranges.

## Methods

### Study area and species

This study was conducted over three years (2014–2016) on the southwest plain of Bylot Island, Nunavut, Canada (73°08′N, 80°00′W), located in Sirmilik National Park. The landscape is dominated by mesic tundra on the uplands and both mesic tundra and wetlands in the lowlands^28^. The mesic tundra is covered with relatively lush vegetation for this latitude, mainly composed of low shrubs (*Salix*, *Vaccinium*) and forbs (e.g. *Cassiope*, *Dryas*) with grasses and mosses^28^. The American Golden Plover and the Common Ringed Plover are both common breeding shorebird species within the study area^27^. The American Golden Plover prefers to breed in the low vegetation of mesic tundra^56^, while the Common Ringed Plover breeds on stony and sandy shores and gravel bars with scarce vegetation along coasts and rivers (Fig. 2)^19,27^. This distinction in main nesting habitats between Golden Plovers and Ringed Plovers (including Semipalmated Plover) is largely shared across populations over their circumpolar distribution^45,47,57–59^.

Over a large portion of its distribution, the Arctic Fox’s abundance and behaviour are known to be affected by small mammal population cycles^28,60,61^, hence affecting predation pressure on alternative prey such as tundra birds^40^. On Bylot Island, predation pressure on bird nests varies according to lemming density and is typically reduced in years of high lemming abundance^39^. Two species are present in the study area: the Collared Lemming (*Dicrostonyx groenlandicus*) and the Brown Lemming (*Lemmus trimucronatus*), which both have cyclic abundances^28^. Live trapping conducted in both mesic and wetland habitats revealed high to moderate abundance of lemmings during the three years of the study (6.5 ± 0.3, 3.6 ± 0.2 and 2.5 ± 0.2 lemmings per hectare, in 2014, 2015 and 2016 respectively^30^). The study area is also characterized by a large colony (about 20 000 pairs) of Greater Snow Geese (*Anser caerulescens atlantica*), which increases the predation risk for shorebird nests^25^.

### Shorebirds nest monitoring

Each summer, nesting plovers were monitored along a 50-km-long coastline. We found nests either opportunistically or through line transect surveys^25^ from mid-June to mid-July. Nests were marked with a 10 cm wooden stick (medical tongue depressor) and a natural object (rock or feather) placed 5 m and 7 m from the nest respectively. We typically visited nests every 3–5 days or every 2–3 days when nearing the estimated hatching date. The incubation stage was estimated for each nest using the flotation method^62^. Incubation lasts 26 days for the American Golden Plover^63^ and 24 days for the Common Ringed Plover^64^. A nest was considered successful if at least one egg hatched or if one of the following criteria were met: (1) <5 mm of residual egg shell was found in the nest material close to the estimated hatching date (2), the nest was hatching (starred or pipped) on the last visit and was empty on the next visit, and (3) the nest was empty on the last visit and the banded adult was later seen with chicks^23^. Shorebirds chicks generally leave the nest within 24 hours of hatching^19^. Predation is the main cause of shorebirds nest failure in our study area^23,65^ and other sources of mortality are marginal (nest abandonment or nest flooding was confirmed in only 3 out of 236 plover nests with known fate).

### Artificial nest experiments

Artificial nest experiments were conducted during the plover incubation period in 2015 and 2016. Each year, a total of 60 paired artificial nests were deployed in suitable habitat used by the two selected plover species: 30 artificial nests were placed on stony shores (nesting habitat of the Common Ringed Plover) and 30 nests in mesic tundra (nesting habitat of the American Golden Plover). The two paired nests were separated by 150–200 m and the distance between artificial nests positioned in each habitat was within the range of distances recorded for real nests on Bylot Island (average distance: 877 m and 547 m for artificial and real nests, ranging from 341 m to 1431 m and from 52 m to 17845 m, respectively). Artificial nests were deployed on 12 July 2015 and 10 July 2016 and were visited after 1, 2, 4 and 6 days. Each artificial nest consisted of four quail eggs (*Coturnix japonica*) placed in a shallow depression in the ground; quail eggs are similar to plover eggs in colour and size. The artificial nests were marked as the real nests (see above), but a nail wrapped in fluorescent tape was hidden beneath the eggs so depredated nests could be located easily. To reduce human scent, all eggs and pieces of material used were handled with latex gloves, and researchers used the sole of their rubber boots to make the nest depression.

The Arctic Fox is the main predator of both real and artificial shorebird nests in our study area^23^. However, artificial nests are more vulnerable to avian predators than real nests, which may result from the absence of parental nest defence and because uncovered artificial nests can be more easily detected by avian predators than nests covered by the incubating adult^23,66^. To examine if habitat-driven patterns in predation risk are affected by the exclusion of avian predators, we conducted another experiment using artificial nests covered with a patch of lichen commonly found in the study area (genus *Bryoria* or *Gowardia*). The lichen covers were maintained on top of the eggs using a wooden stick inserted in the ground in the middle of the nest. We deployed 20 paired covered artificial nests on 28 June 2016 (20 nests in stony shores and 20 in mesic tundra), and visited them after 2, 6 and 15 days of exposure. Using motion-triggered cameras^23^, we confirmed that covered artificial nests were only depredated by the Arctic Fox in our study area (Bêty and Léandri-Breton, unpublished data, N = 82 depredated nests monitored with cameras in 2015 and 2016). Covered nests also allowed us to control for potential differences in quail egg crypsis associated with habitat types.

Field techniques were approved by Université du Québec à Rimouski Animal Care Committee and field research was approved by the Joint Park Management Committee of Sirmilik National Park of Canada.

### Statistical analyses

For real shorebirds nests, we compared the daily nest survival of the two plover species using the logistic-exposure method^67^. Year was included as a random factor to control for inter-annual variations. The logistic-exposure method is a generalized linear model with a binomial response distribution (1 when the interval nest fate is a success and 0 when depredation occurred) using a logit link function to account for variations in the length of observation intervals. This method is advantageous as it allows the inclusion of random effects (e.g. “year”) and requires no assumptions about when nest losses occur. We assumed a constant daily nest survival during the nesting period. For graphical representation, daily nest survival was estimated through a logistic-exposure model per species and per year. The nest success probability over the entire incubation period was obtained by raising the daily nest survival to the power of the mean incubation period of each species (26 days for the American Golden Plover and 24 for the Common Ringed Plover).

We used Cox proportional hazards regression (R package “survival” 2.40-1^68^) to assess the effect of habitat types on the survival of artificial nests. Cox model tests for a relationship between Kaplan-Meier survival estimates, a nonparametric statistic commonly used to estimate survival over time, and explanatory variables^69,70^. Cox model is very appropriate for artificial nests because it allows for right-censoring data when nests survive past the end of the experiment. Year was treated as a random variable. The proportion hazards assumption was verified by calculating the correlation between scaled Schoenfeld residuals and survival time. For visualization purposes, predation risk was estimated by fitting a Kaplan-Meier survival probability curve for each species and “year” without random effect. All analyses were carried out in R version 3.2.2^71^.

## Data Availability

The dataset analyzed during the current study is available on the public repository Dryad at 10.5061/dryad.4xgxd254v.
